# Hierarchically mesoporous CuO/carbon nanofiber coaxial shell-core nanowires for lithium ion batteries

**DOI:** 10.1038/srep09754

**Published:** 2015-05-06

**Authors:** Seok-Hwan Park, Wan-Jin Lee

**Affiliations:** 1 Faculty of Applied Chemical Engineering, Chonnam National University, Gwangju 500-757, Korea; 2 Alan MacDiarmid Energy Research Institute, Chonnam National University, Gwangju 500-757, Korea

## Abstract

Hierarchically mesoporous CuO/carbon nanofiber coaxial shell-core nanowires (CuO/CNF) as anodes for lithium ion batteries were prepared by coating the Cu_2_(NO_3_)(OH)_3_ on the surface of conductive and elastic CNF via electrophoretic deposition (EPD), followed by thermal treatment in air. The CuO shell stacked with nanoparticles grows radially toward the CNF core, which forms hierarchically mesoporous three-dimensional (3D) coaxial shell-core structure with abundant inner spaces in nanoparticle-stacked CuO shell. The CuO shells with abundant inner spaces on the surface of CNF and high conductivity of 1D CNF increase mainly electrochemical rate capability. The CNF core with elasticity plays an important role in strongly suppressing radial volume expansion by inelastic CuO shell by offering the buffering effect. The CuO/CNF nanowires deliver an initial capacity of 1150 mAh g^−1^ at 100 mA g^−1^ and maintain a high reversible capacity of 772 mAh g^−1^ without showing obvious decay after 50 cycles.

The electrically active transition metal oxides (M_x_O_y_, M = Ni, Co, Cu, Fe, Mn) such as CuO have attracted much attention as anode materials to substitute graphite in Lithium ion batteries (LIB) due to their high theoretical reversible capacity (674 mAh g^−1^) on the basis of their unique conversion mechanism, (*MO + 2Li*^*+*^ + *2e*^*−*^* = Li*_*2*_*O + M*), low material cost, chemical stability, nontoxicity and plenty^1^,^2^,^3^,^4^,^5^,^6^,^7^,^8^,^9^,^10^,^11^. However, the CuO has mostly poor kinetics and unstable capacity during the cycling, primarily because of the low conductivity and the pulverization due to large volume expansion during cycling, leading to rapid capacity fade^8^,^9^,^10^,^11^. To overcome these problems, CuO has been designed in a variety of morphology such as nanowire arrays^12^, nanocages^13^, CuO/graphene composites^10^, CuO/CNT composites^9^, CuO/carbon composite nanowire^14^, and other recent researches^15^,^16^,^17^,^18^,^19^,^20^,^21^,^22^,^23^. Nevertheless, it is hard to appropriately control the capacity decay by lithiated CuO volume expansion.

The effective strategy to increase the performance of anode materials is deeply dependent on the modification of morphology. Better nanostructured composites lead to improved electrochemical performance with good structural stability, high surface area with high mesoporosity, good electrical contact between electrode and electrolyte, and increased electrical conductivity. Electrophoretic deposition (EPD) used in this study as a means of preparing excellent nanostructured composites is a facile synthetic technique to coat Cu_2_(NO_3_)(OH)_3_ nanoparticles from the Cu(NO_3_)_2_ ethanol solution on the surface of CNFs as a cathode under an applied electric field^24^,^25^,^26^. This useful technique is remarkably unique and novel that has not been conducted for the CuO/CNF system previously. Under an applied electric field, the charged ions in a solution move toward the oppositely charged electrode by the phenomenon electrophoresis. After the charged ions accumulate at the electrode, they deposit as proper structures by controlling the rate of mass transfer. The deposited electrode makes crystallization by a heat-treatment process. The EPD method offers 3D hierarchically porous CuO/CNF coaxial shell-core nanowires. The CuO shell with abundant inner spaces provides the excellent rate capability. The mesoporous structures with abundant inner spaces enables the electrolyte to access easily to the CuO anode material. Without the role of CNF core, the radial compression by lithiated CuO during cycling results in large volume expansion. The metal oxide such as CuO represents the inelastic nature, whereas the CNF shows the elastic characteristic with high elastic modulus^15^,^28^. During cycling, the elastic CNF core plays an important role in protecting volume expansion along with the radial compression of lithiated CuO shell by creating the cushioning effect. Moreover, the conductive CNF core with 1D pathway facilitates the electron transfer, leading to the improvement of charge transfer.

An aim in this study is to design a novel 3D coaxial CuO/CNF composite nanowires to achieve high rate capability and good electrochemical retention without obvious decay, at the same time. The 3D coaxial CuO/CNF nanowires are prepared by directly coating with Cu_2_(NO_3_)(OH)_3_ nanoparticles on CNF through an electrophoretic deposition (EPD), and the subsequent heat treatment.

## Results and discussion

**Figure 1:**
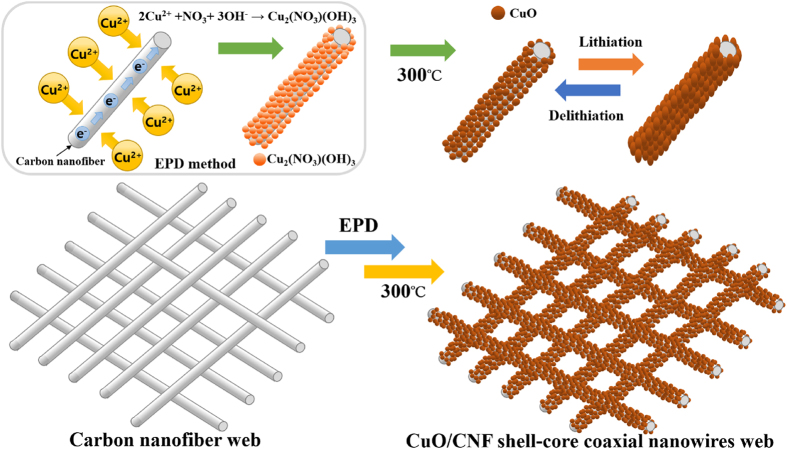
The fabricating process of CuO/CNF. The process of Cu_2_(NO_3_)(OH)_3_ deposition on the surface of CNF through a facile EPD method.

The process of Cu_2_(NO_3_)(OH)_3_ deposition on the surface of CNF through a facile electrophoretic deposition (EPD) method is shown in Fig. 1. When an electric field is applied, the Cu^2+^ ions in Cu(NO_3_) _2_·6H_2_O ethanol solution transfer toward the surface of a one-dimensional (1D) CNFs cathode, and then Cu^2+^ ions are adsorbed on the surface of CNF, forming the positively charged CNF-Cu^2+^. At the same time, the NO_3_^−^ ions of copper nitrate are electrochemically reduced with H_2_O, and then the produced OH^−^ ions and the residual NO_3_^−^ ions move toward CNF-Cu^2+^ without diffusing into the bulk solution. Finally, Cu_2_(NO_3_)(OH)_3_ on the surface of CNF is deposited to rapidly react Cu^2+^ with both NO_3_ and OH^−^. The mechanism to be deposited Cu_2_(NO_3_)(OH)_3_ to the surface of CNF cathode is as follows^29^:
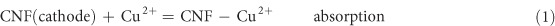

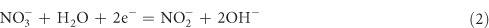

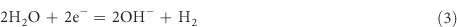

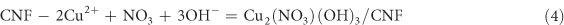

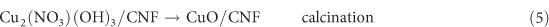


**Figure 2:**
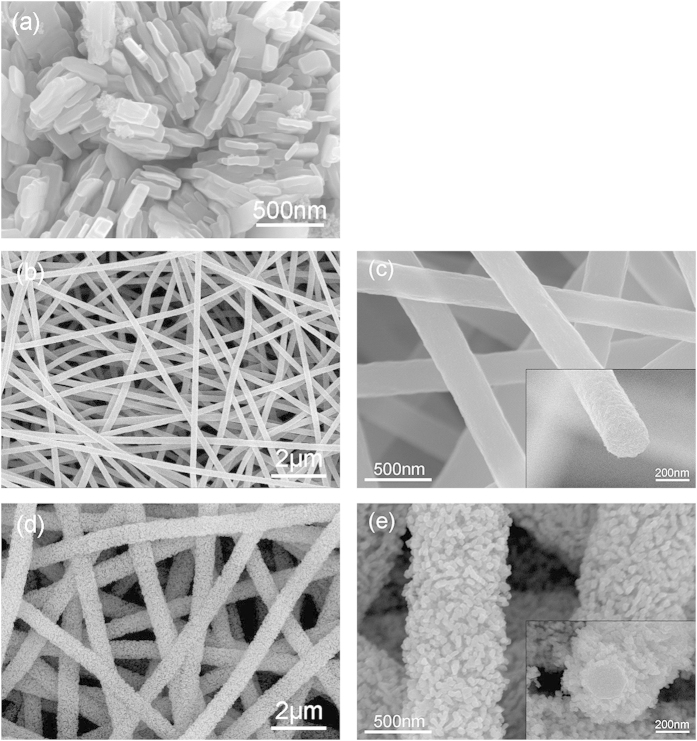
SEM images for (**a**) CuO, (**b-c**) CNF, and (**d-e**) CuO/CNF.

Figure 2 shows the SEM images for the surface of CuO powder, pure CNF, and CuO/CNF. In Fig. 2a, the CuO powder has the rectangular-like shape, which the particles range from 100 nm to 1 μm in length. In Fig. 2b and c, pure CNF represents the woven network structure offering diffusion pathway between about 250 nm CNFs of about 250 nm in diameter. The CNF with 1D morphology has the coarse surface suitable for coating the precursor of CuO. The CNFs is well-known to have high elastic modulus, which can absorb the radial compression giving rise to the volume expansion during cycling^27^,^28^. As for Fig. 2d and e, the CuO/CNF prepared by EPD method display the 3D coaxial shell-core network structure interconnected with hierarchically porous nanowires. In Fig. 2e, the CuO shell on the surface of the CNF represents the particle-stacked morphology as a result of vertically grown CuO nanoparticles of around 20 nm in diameter. The growth of the nanoparticle-stacked morphology would be the cause of Cu_2_(NO_3_)(OH)_3_ adsorption on the π-electrons of the CNFs. The CuO shell of about 110 nm thick surrounds by coating with nanopartilces outward on the surface of CNF core of 220 nm in diameter. This nanoparticle-stacked shell and the interlayers between CuO/CNF nanowires provide a highly porous 3D structure with a large surface area and abundant inner spaces. This morphology offers tremendous channels for the facile electrolyte flow, and the high contact area between the electrolyte and CuO. This characteristics facilitates mass transfer and charge transfer in enhancing the electrochemical performance.

**Figure 3:**
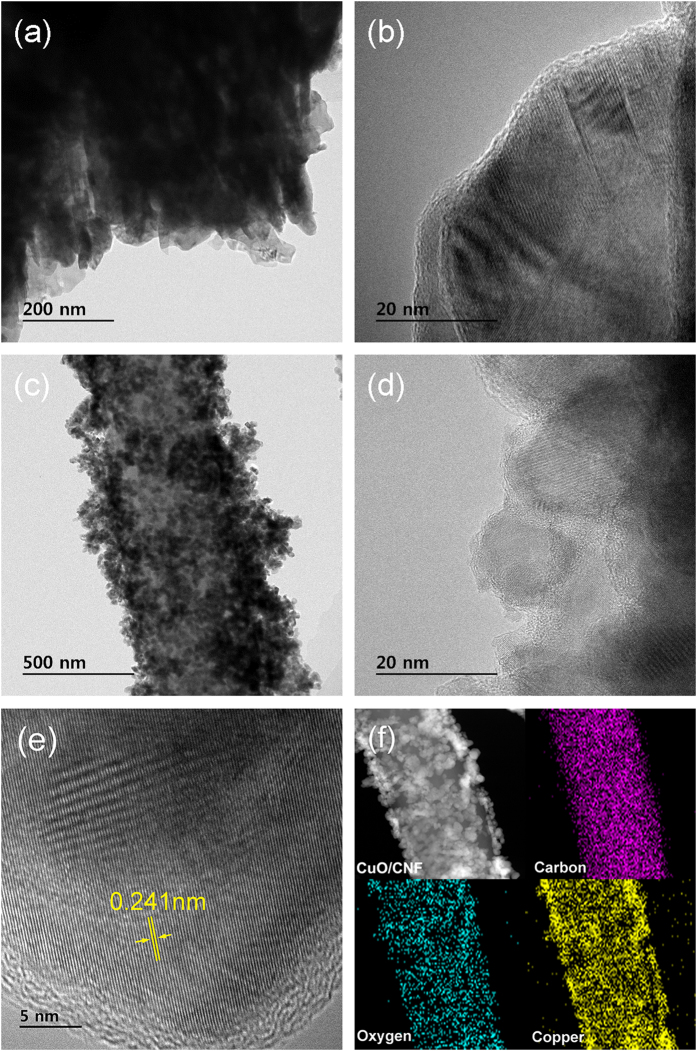
TEM images for (**a,b**) CuO and (**c-e**) CuO/CNF; STEM image and EDAX elemental mapping of (f) CuO/CNF.

Figure 3 shows TEM images for the CuO powder, and CuO/CNF. In Fig. 3a and b, the CuO powders consisting of polygon nanoparticles are tightly packed in a bundle-like shape. The CuO nanoparticles are in the range of about 50 to 100 nm in size. The CuO/CNF shown in Fig. 3c possesses 3D coaxial morphology consisting of the CNF core and the CuO shell. The CuO shell of about 150 nm thick surrounds the CNF in a diameter 220 nm, and the CuO nanoparticles in the CuO shell appear to pile up outward from the CNF, offering mesoporous structure of CuO shell. In Fig. 3d, it is observed that the stacked nanoparticles in the CuO shell represent spherical shape, offering a highly mesoporous structure caused by pores between the interconnected spherical CuO nanoparticles. The spherical CuO nanoparticles are in the range of about 15 to 20 nm in diameter. In the CuO/CNF shell-core nanowires, the CuO shell shows the inelastic characteristic as a serious problem incurring radial volume expansion during cycling, while the CNF has the elastic characteristic with high modulus absorbing the radial volume expansion of the lithiated CuO^27^,^28^. The HR-TEM image of the CuO shell is shown in Fig. 3e. The distance between adjacent planes in CuO particle represents approximately 0.241 nm, corresponding to the lattice spacing of the 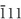 planes for CuO. In Fig. 3f, the characteristics of core-shell structure for the CuO/CNF are separately confirmed by the scanning transmission electron microscopy (STEM) and energy dispersive X-ray analysis (EDX) elemental mappings.

**Figure 4:**
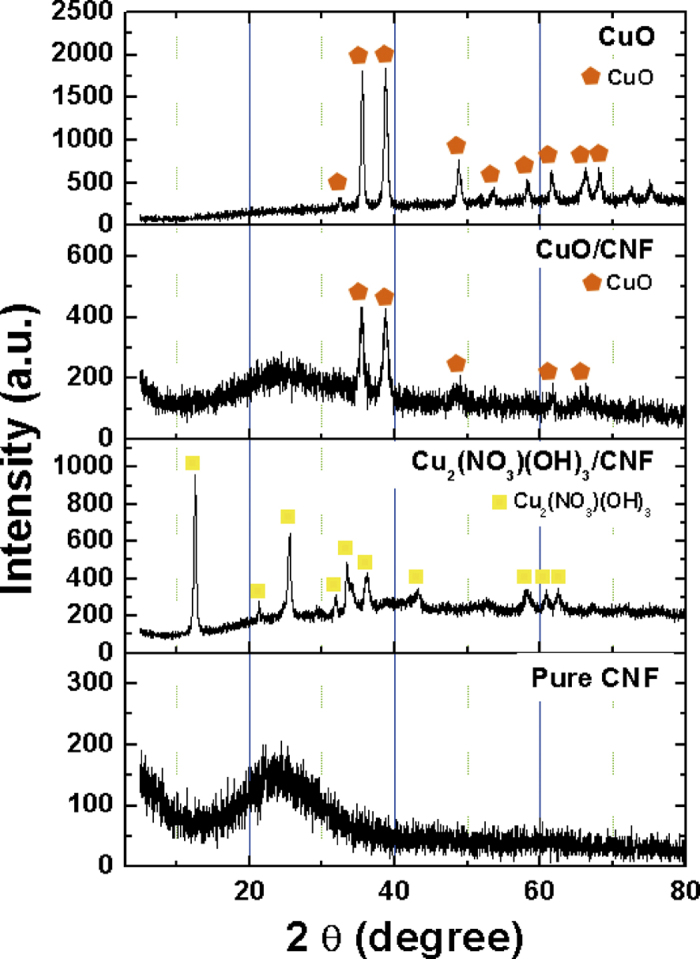
XRD patterns of CNF, Cu_2_(NO_3_)(OH)_3_/CNF, CuO and CuO/CNF.

Figure 4 represents the XRD patterns of CuO powder, pure CNF, and CuO/CNF. The CNF has the broad peak at around 24 ^o^, which shows the typical amorphous structure. All the peaks of Cu_2_(NO_3_)(OH)_3_/CNF are indexed as a single phase of Cu_2_(NO_3_)(OH)_3,_ (JCPDS No. 45-0594). The major diffraction peaks of CuO powder are formed at (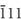) and (1 1 1) planes of the monoclinic CuO phase (JCPDS No. 05-0661), which is no peaks of Cu_2_O or Cu. In the CuO/CNF, all of the peaks the peaks of CuO/CNF nearly coincide with those of pure CuO particle, which represents that the Cu_2_(NO_3_)(OH)_3_ adsorbed on the surface of CNF is well transformed with CuO/CNF composites. The peaks of CuO/CNF are weaker and wider than those of CuO powder, indicating that the Cu_2_(NO_3_)(OH)_3_ is slowly deposited as a nanoparticles on the surface of CNF due to slow mass transfer induced by low 10 V DC in EPD process.

**Figure 5:**
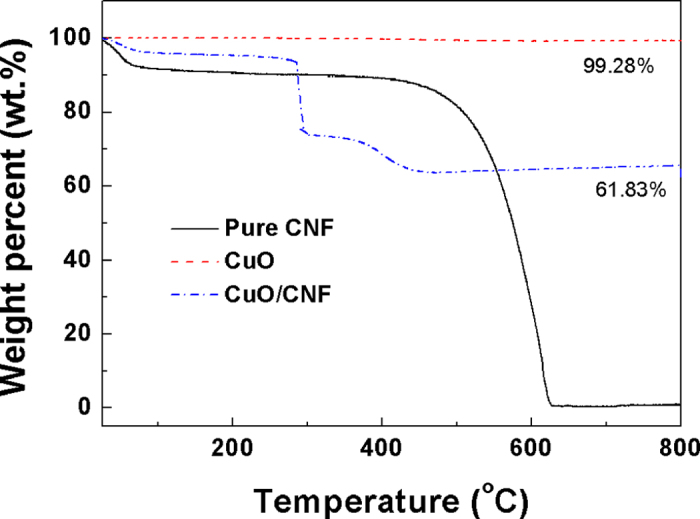
TGA thermograms of CNF, CuO and CuO/CNF in air.

Figure 5 shows the TGA results of CuO powder, pure CNF, and CuO/CNF in air. The CuO powder has almost no weight loss except for the loss of water due to the formation of only metal oxide. For pure CNF, the weight loss by the evaporation of water occurs in the rage of 25 to 200 ^o^C, and the complete decomposition arises at about 630 ^o^C. As for CuO/CNF, the degradation occurs through three steps. The first weight loss corresponds with the evaporation of water and solvent in the range of 25 to 200 ^o^C. The second weight loss ascribes to the degradation of CNF side chain between 200 and 300 ^o^C. The last weight loss attributes to the complete decomposition of CNF main chain from 300 to 600 ^o^C, indicating that the CuO content remains 61.8 wt.% in CuO/CNF. This means that the weight ratio of CuO to CNF is 61.8 : 38.2.

**Figure 6:**
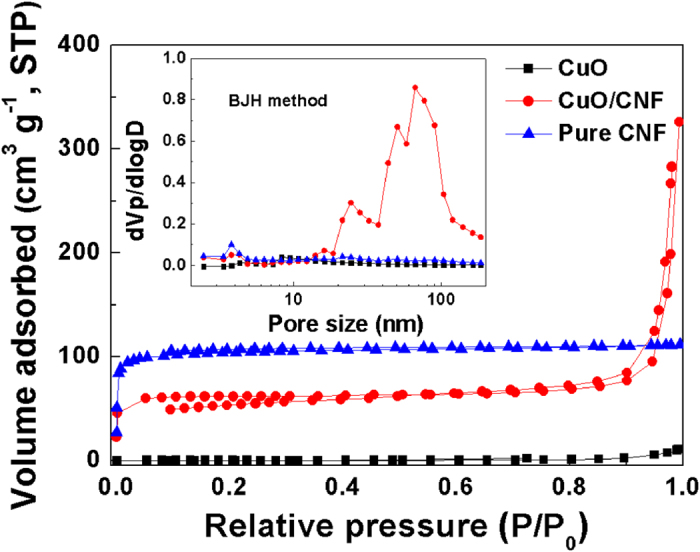
N_2_ sorption isotherms and pore size distribution of CNF, CuO and CuO/CNF.

Figure 6 represents the isotherms of nitrogen adsorption-desorption for the CuO powder, pure CNF, and CuO/CNF. The adsorption for CuO powder hardly occurs in the range of all the relative pressure, indicating that there is little porous structure. For pure CNF, the adsorption is saturated at a low relative pressure of approximately 0.01, indicating the well-microporous structure as a typical type I isotherm. The existence of this steep knee points out the occurrence of a narrow micropore size distribution. As for CuO/CNF, the hysteresis loop in the isotherm starts to form at a relative pressure of approximately 0.6, and then becomes steep at a relative pressure of around 0.85. The hysteresis phenomenon in the adsorption/desorption process is occurred due to the nanoparticles-stacked morphology of CuO shell. This is why the coaxial CuO/CNF is coated the mesoporous CuO shell on the surface of microporous CNF core.

The inset graph of Fig. 6 shows the pore size distributions by BJH for the CuO powder, pure CNF, and CuO/CNF. The CuO powder does not show pore size distribution because there is little porous structure. Pure CNF shows only microporous morphology without the existence of mesoporosity. For the CuO/CNF, the pores of three groups are observed: (i) small mesopore ranging from 3 to 5 nm, (ii) large mesopore ranging from 15 to 20 nm, and (iii) macropore ranging from 50 to 120 nm. The small and large mesopores are originated from porous nature of nanoparticle-stacked CuO structure, and the macropores are formed by the interlayers between nanowires. This highly hierarchical nanoparticle-stacked porous morphology with abundant inner spaces promote the facile diffusion of electrolytes into the inner region of CuO/CNF to reduce the diffusion resistance, and provides more active sites for redox reaction.

Table 1 represents the pore characteristics of the CuO powder, pure CNF, and CuO/CNF, which are calculated by the BET equation and the BJH method from the nitrogen adsorption-desorption isotherms at 77 ^o^K. The CuO powder has nearly undeveloped morphology, which the total surface area is only 5 m^2^ g^−1^. The pure CNF is composed of nearly microporous morphology, representing that the specific surface area, microporopus surface area, total pore volume, and average pore diameter are 378 m^2^ g^−1^, 367 m^2^ g^−1^, 0.17 cm^3^ g^−1^, and 1.82 nm, respectively. For the CuO/CNF composite, the specific surface area, mesoporopus surface area, total pore volume, and average pore diameter are 244.7 m^2^ g^−1^, 58.4 m^2^ g^−1^, 0.46 cm^3^ g^−1^, and 7.57 nm, respectively. This evidence means the hierarchically coaxial CuO/CNF composite is consisted of the morphology coated with the mesoporous CuO shell on the surface of the microporous CNF core, which is suitable for enhancing the electrochemical rate capability. The specific surface area of CuO/CNF appears to be low compared to CNF because the micropores of CNF are covered by the CuO shell.

**Figure 7:**
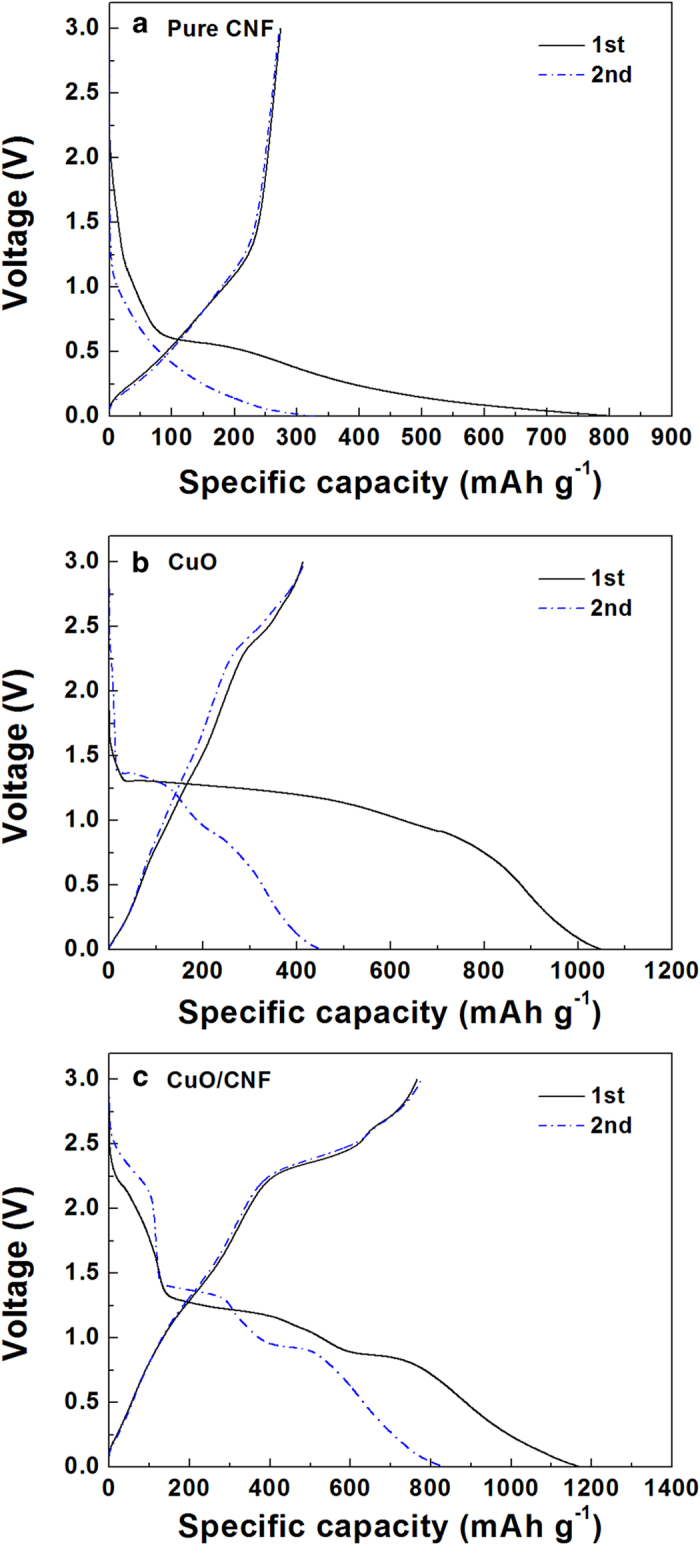
Voltage profiles of (**a**) CNF, (**b**) CuO, and (**c**) CuO/CNF at 100 mA g^–1^ in 1 M LiPF_6_/EC/DMC.

Figure 7 displays the initial and the second charge and discharge profile of the CuO powder, and CuO/CNF composite electrodes with the voltage ranging from 0.02–3 V at a current density of 100 mA g^−1^ using a cell assembled Li metal. For pure CNF shown in Fig. 7a, the specific capacity is much lower than other two samples, although its specific capacity is similar to that of carbonaceous material like the graphite. For CuO powder of Fig. 7b, the initial charge and discharge capacities of the CuO powder are 1051 and 413 mAh g^−1^, representing a low coulombic efficiency of 39%, and the second charge and discharge capacities 452 and 416 mAh g^−1^, respectively. The CuO electrode represents high irreversible capacity during the initial cycle, followed by the abrupt capacity decay after the second cycle. For the CuO/CNF of Fig. 7c, the CuO/CNF electrode exhibits a high irreversible capacity during the initial cycle and then the capacity is stabilized during the subsequent cycle. The electrochemical conversion mechanism of the reaction between lithium and CuO is followed by CuO + 2Li^+^ + 2e^−^ → Li_2_O + Cu. During the initial Li ion charge (insertion) reaction, an obvious sloping voltage for CuO/CNF is observed from 2.5 to 1.3 V due to the formation of solid-electrolyte interface (SEI) film. A well-defined voltage plateau at around 1.3 V is contributed from the main lithiation reaction of CuO/CNF for the formation of solid solution, Li_x_Cu_1−x_^2+^Cu_x_^+^O^30^. The voltage plateau at about 1.3 V reflects the Li ion charge (insertion) reaction. Similar to the CV results, the lithiation plateau moves to a higher voltage of around 1.4 V in the second cycle, which implies the electrochemical reversibility by the easy polarization after the initial charge cycle. The discharge curves have two slope plateaus in the ranges of about 2.2 – 2.45 V and 2.7 V, corresponding to the formation of CuO from Cu, Cu_2_O, and Li_2_O. In the initial cycle, the charge and discharge capacities were 1150 and 766 mAh g^−1^, respectively. The irreversible capacity of 67% in the initial cycle is attributed to the formation of SEI films and on the surfaces of the CuO/CNF, and the intercalation of lithium ions into abundant inner space of woven network interconnected with 3D coaxial CuO/CNF nanofiber. From the second charge-discharge curves, the plateaus are not clear caused by low hysteresis of potential, representing that the reaction appears to be more reversible. The coulombic efficiency from the second cycle increases steeply to 93%, showing that the charge and discharge capacity are 832 and 777 mAh g^−1^, respectively.

**Figure 8:**
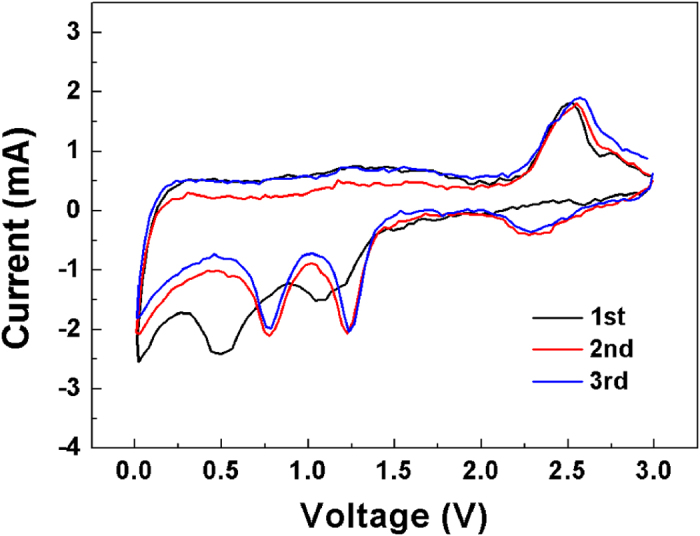
Cyclic voltammograms of CuO/CNF at a scanning rate of 0.2 mV s^–1^ in 1 M LiPF_6_ EC/DMC electrolyte.

The cyclic voltammograms (CV) of CuO/CNF composite electrodes in the range of 0–3 V at 0.2 mV s^−1^ scan rate is shown in Fig. 8. For the first cathodic scan, the broad peak at 1.1 V is due to the formation of Cu_2_O phase and and the formation of a partly reversible SEI layer (2CuO + 2Li^+^ → Cu_2_O + Li_2_O)^30^. Another peak around 0.5 V is related to the further decomposition of Cu_2_O phase into Cu and amorphous Li_2_O (Cu_2_O + 2Li^+^ → 2Cu + Li_2_O). Two peaks positioned at about 2.5 V and 2.7 V in the first anodic scan, which corresponds to the formation of CuO (Cu + Li_2_O → CuO + 2Li^+^) and the oxidation of Cu_2_O into CuO (Cu_2_O + Li_2_O → 2CuO + 2Li^+^), respectively^30^,^31^,^32^,^33^. From the subsequent cycles, the cathodic peak becomes smaller, which suggests the formation of SEI and the irreversible reaction (CuO + 2Li^+^ → Cu + Li_2_O), and the peak shifted to higher potential due to the easy polarization with reversibility. The variations of main peaks to higher voltage with the increase of cycles continue are deeply dependent on to the hierarchically porous shell-core structured CuO/CNF with high surface area.

**Figure 9:**
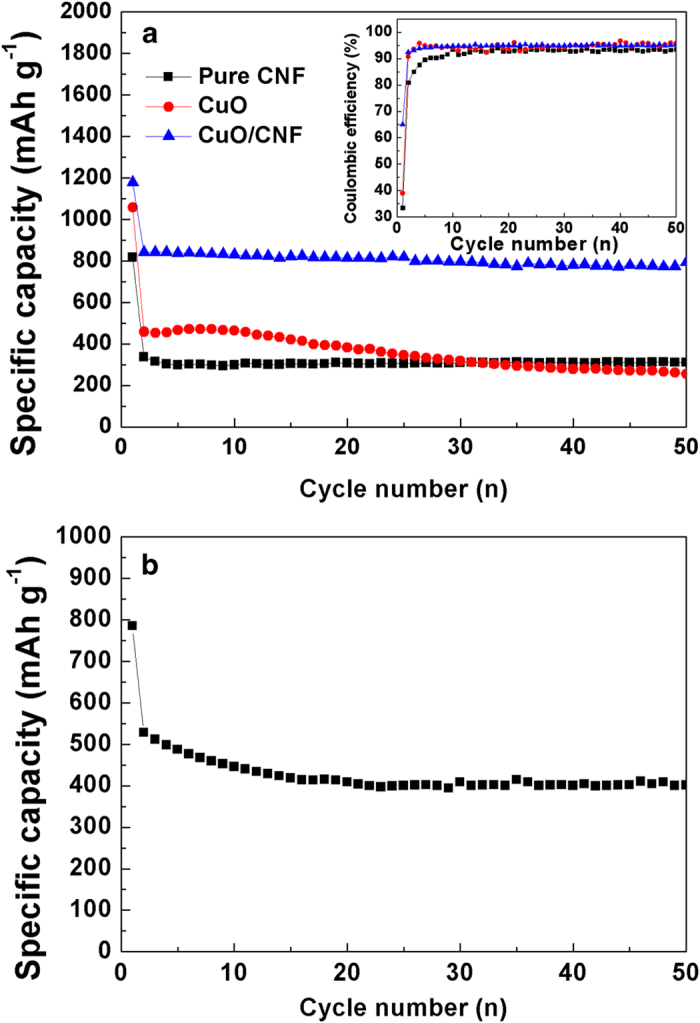
(**a**) Cycle performance of CNF, CuO, and CuO/CNF at 100 mA g^–1^, and (**b**) capacity retention of CuO/CNF at 1000 mA g^–1^ in 1 M LiPF_6_/EC/DMC.

Figure 9a shows the cycle performance of the CuO powder, pure CNF and CuO/CNF. The specific capacity for CuO powder reaches 1051 mAh g^−1^ in the initial cycle eventually leveling off to 290 mAh g^−1^ in the 50^th^ cycle due to fatal volume changes, which is even lower than near the value of the pure CNF. The coulomb efficiencies for the initial cycle are ranked as: CuO/CNF (65%) > CuO (39%) > pure CNF (34.5%). The PVDF is generally used in preparing the electrodes of lithium ion batteries. In this work, the poly (acrylic acid) (PAA) is used to offer the facile adhesion between active electrode materials. The PAA as a binder may lead to the slight decrease in coulombic efficiency of CNF, CuO, and CuO/CNF owning to high adhesion strength^34^. The CuO/CNF represents the excellent capability and electrochemical stability at the same time, which represents more than 830 mAh g^−1^ after the second cycle without an obvious capacity fading except for an initial capacity of 1150 mAh g^−1^. The specific capacity of CuO/CNF is much higher than the theoretical capacity of 559 mAh g^−1^ of CuO/CNF. Theoretical capacity of CuO/CNF is calculated as follow: theoretical capacity (TC) of CuO/CNF = TC of CuO × weight% of CuO + TC of graphite × weight% of graphite = 674 × 61.8% + 372 × 38.2% = 559 mAh g^−1^. The weight% of CuO/CNF obtained from the result of TGA is used in calculating theoretical capacity of CuO/CNF. In addition, the CuO/CNF still show good reversible capacity (400 mAh g^−1^) after 50 cycles despite high current density (1000mA g^−1^) as shown in Fig. 9b. The reasons for high capability and excellent retention are as follows. Firstly, the 3D coaxial CuO/CNF connected with CuO shell on the surface of CNF creates the excellent retention without fading for cycling. During cycling, the CuO shell compresses the surface of elastic CNF core toward the radial direction through inelastic flow because the large volume expansion of the lithiated CuO in the shell is mostly in the radial direction^27^,^28^. Because the elastic CNF core offers the buffering effect against the inelastic CuO shell, 3D coaxial CuO-CNF shell-core morphology protects the battery failure coming from volume variation by the inelastic CuO shell without the fading of capacity. Secondly, both abundant inner spaces within nanoparticle-stacked CuO shell and a lot of pores between interlayers of nanowires not only offers tremendous channels for the facile electrolyte flow, but also induces excellent contact between the electrolyte and electrode. This porous morphology by 3D coaxial CuO/CNF shell-core nanowires promotes mass transfer and charge transfer in enhancing the electrochemical specific capacity. Thirdly, the CNF core with 1D structure leads to the increased electrical conductivity and mechanical stability to CuO/CNF nanowires. The electrical networking makes electron transfer easier by increasing the electrical conductivity. The mechanical networking toughens the structural stability of nanoparticle-stacked CuO shell on the surface of CNF.

**Figure 10:**
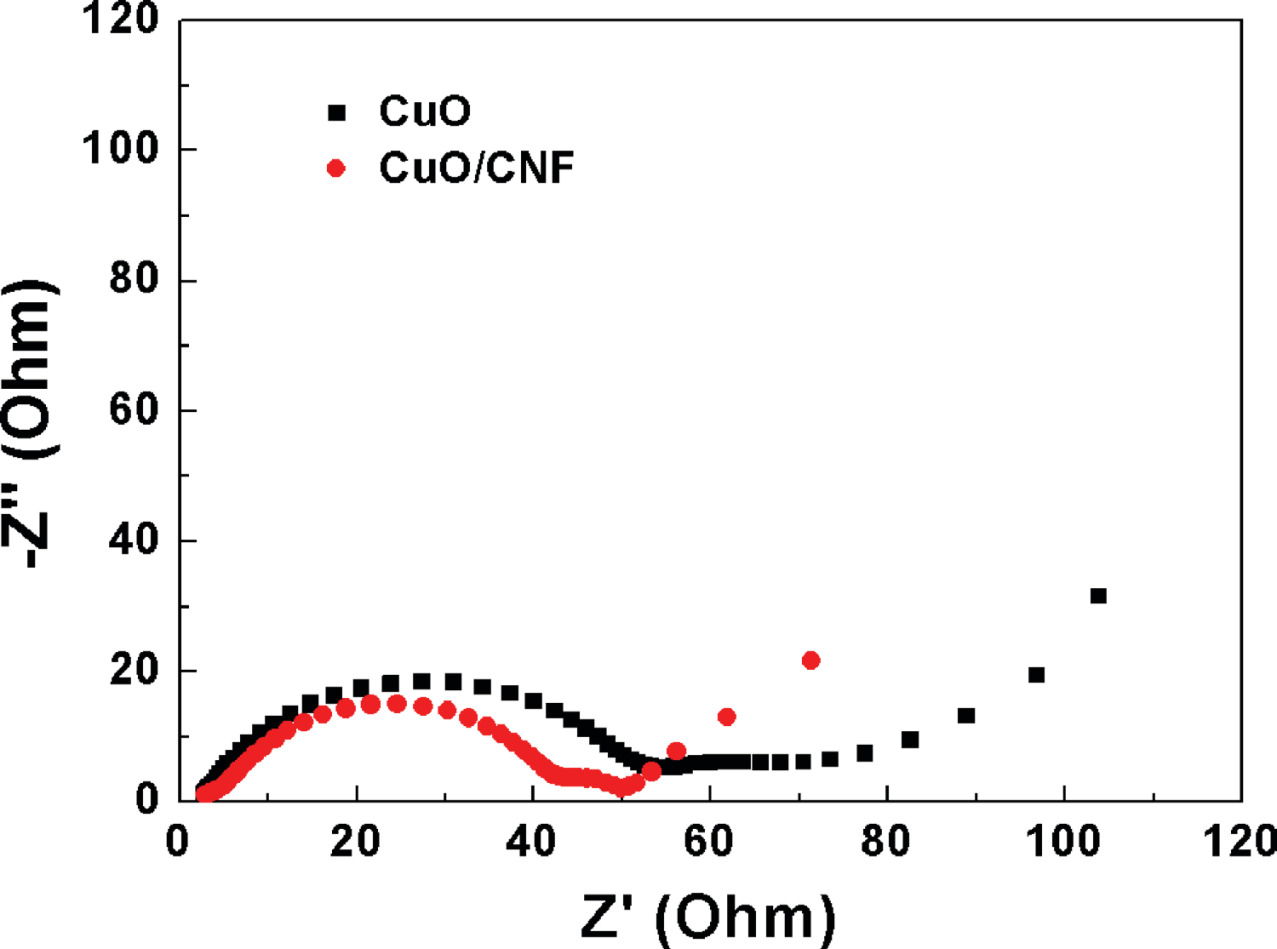
AC impedance spectra of CuO and CuO/CNF in 1 M LiPF_6_/EC/DMC.

Figure 10 shows the change in electrochemical impedance spectroscopy (EIS) curve by the Nyquist plots in the range of 100 kHz to 10 mHz for CuO powder, and CuO/CNF electrodes. The internal resistance (R_Ω_) is placed in the intercept of the semicircle in the high frequency region at the real axis. The internal resistances of both CuO powder and CuO/CNF electrodes is almost the same as around 2.0 Ω, because there is little difference originated from the intrinsic electrical resistance of the active materials, the electrolyte resistance, and the contact resistance at the interface between the active material and current collector. The charge-transfer resistance (R_ct_) lies in the semicircle at low frequency. The charge-transfer resistances of the CuO powder and CuO/CNF electrodes are 53 and 42 Ω, respectively. The charge-transfer resistance of the CuO/CNF electrode is smaller than that of CuO powder electrodes, due to the facile lithium ion transfer by abundant inner spaces in CuO shells, the increased electrical conductivity by CNFs, and the structural stability by 3D coaxial core-shell morphology. These factors benefits to reduce the ion intercalation distance, facilitate charge transfer, and reduce the resistance.

**Figure 11:**
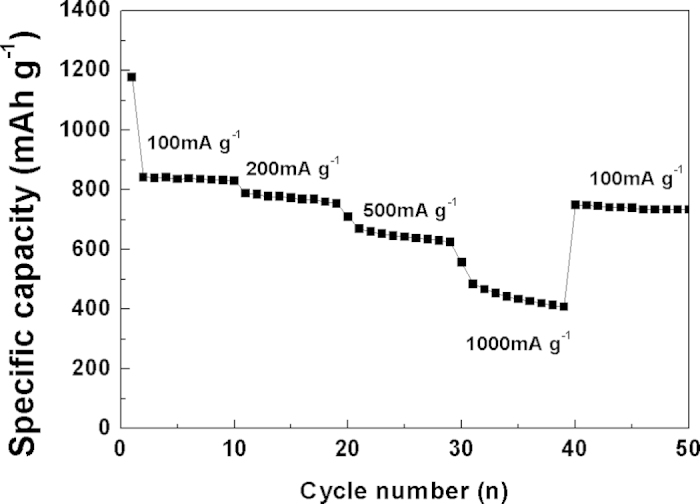
Rate performance of CuO/CNF at various current densities from 100 to 1000 mA g^–1^.

Figure 11 shows the capacity of CuO/CNF at various current densities. The specific capacities show at 832, 789, 669, and 485 mAh g^−1^ as the current densities increase 100, 200, 500, and 1000 mA g^−1^, respectively. Afterwards, the specific capacity at the current density of 100 mA g^−1^ delivers approximately 750 mAh g^−1^ (recovery efficiency: 90%) representing the recovery efficiency of around 90%, representing excellent reversibility of the conversion reaction between CuO and Cu. The reasons the CuO/CNF composite has both exceptional retention and excellent rate capability is as follows. Firstly, the coaxial CuO/CNF shell-core morphology with high surface area facilitates Li insertion and extraction, and ion transfer by offering a smaller resistance and shorter diffusion pathways. This hierarchically mesoporous nanoparticle-stacked CuO morphology on the surface of CNF generates the excellent rate capability. Secondly, the enhancement of electrical conductivity and mechanical networking by the 1D CNF facilitates redox reaction, and enforces the mechanical stability. Thirdly, the elastic CNF core prohibits radial volume expansion coming from the inelastic CuO shell by the buffering effect, inducing the exceptional electrochemical stability.

## Conclusions

Hierarchically mesoporous coaxial CuO/CNF shell-core nanowires with 3D sturcture as anode materials for LIBs were prepared via electrophoretic deposition (EPD) on the surface of CNF, and the subsequent heat treatment, featuring hierarchically mesoporous nanoparticle-stacked CuO shell on the surface of CNF core. The CuO shell stacked with about 20 nm nanoparticles grows radially toward the surface of CNF core. The CuO/CNF nanowires possess not only the abundant inner spaces in CuO shells but also a lot of pores between the interlayers of CuO/CNF nanowires, easily penetrating the electrolyte to enhance the electrochemical performance. The CuO/CNF composites deliver an initial capacity of 1150 mAh g^−1^ at 100 mA g^−1^ and retains a high reversible capacity of 772 mAh g^−1^ after 50 cycles without showing obvious decay. The reasons for both the excellent retention and good rate capability for the CuO/CNF composites as follows: (i) easy lithium insertion/extraction and facile lithium ion transfer by 3D coaxial core-shell CuO/CNF composites with high surface area, creating electrochemical rate capability, (ii) the protection of radial volume expansion of the inelastic CuO shell by the buffering effect of elastic CNF core, generating the electrochemical stability, and (iii) the enhancement of electrical conductivity by 1D CNF, facilitating the electron transfer.

## Methods

### Preparation of Carbon Nanofibers

The polymer solution for electrospinning was prepared by dissolving 10 wt.% polyacrylonitrile (PAN, MW = 150,000, Aldrich Chemical Co) in N, N-dimethylformamide (DMF), followed by gently stirring for 24 h at 60 °C to obtain a homogeneous solution. The electrospinning process was conducted out by the system of the previous work, which is installed with a power supply (NT-PS-35K, NTSEE, Korea) with a controllable high voltage^35^,^36^,^37^. The polymer solution was placed in a 30 ml syringe with a capillary tip (ID = 0.5 mm). The anode of the high voltage power supply was clamped to a syringe needle tip, and the cathode was connected to a metal collector. The electrospun fibers were collected on aluminum foil, which was wrapped around a metal drum rotating at approximately 300 rpm. The applied voltage was 20 kV, the distance between the tip and the collector was 18 cm, and the flow rate of the spinning solution was 1 ml h^−1^ 35,36,37. The electrospun fibers were stabilized by heating to 280 °C at a rate of 1 °C min^−1^ in air, and holding them for 1 h. Finally, the CNF was prepared by carbonizing the stabilized fibers for 1 h after increasing to 1000 °C at a rate of 5 °C min^−1^ under nitrogen.

### Preparation of CuO powder

The CuO powder was prepared by calcining 0.5 g of Cu(NO_3_)_2_·6H_2_O for 1 h after increasing to 350 °C at a rate of 5 °C in air, and the subsequent cooling down to room temperature. The mechanism of reaction is as follows: Cu(NO_3_)_2_ → CuO↓ + 2NO_2_↑ + O_2_↑^38^.

### Preparation of CuO/carbon nanofiber shell-core coaxial nanowires

Electrophoretic deposition (EPD) is a facile method to coat a precursor of CuO nanoparticles on the surface of CNF under an applied electric field. The CNF were used as a cathode, and the Pt wire was an anode. Two electrodes spaced 5 cm apart in an electrolytic cell were immersed in Cu(NO_3_)_2_·6H_2_O ethanol solution. After applying the potential of 10 V for 4 h to an electrolytic cell, the product was washed several times with ethanol, and then dried completely at room temperature. Finally, the CuO/CNF shell-core nanowires were prepared by annealing Cu_2_(NO_3_)(OH)_3_/CNF obtained by EPD technique at 300 ^o^C for 2 h under air atmosphere.

### Characterization

The morphologies of the CuO powder, pure CNF and CuO/CNF were observed by using field emission scanning electron microscope (FE-SEM, S-4700, Hitachi, Japan). The elemental mappings, particle size, and the dispersion degree of CuO on the surface of CNF were verified using transmission electron microscope (TEM, TECNAI F20, Philips, Netherlands) in the Korean Basic Science Institute (KBSI, Gwangju center). The weight loss of CuO/CNF was measured by thermogravimetric analysis (TGA, Shimadzu, TA-50, Japan). The crystallization results for pure CNF, CuO powder and CuO/CNF were analyzed by the X-ray diffraction (XRD, D/MAX Uitima III, Rigaku, Japan). Electrochemical charge–discharge behaviors were examined using coin cells (type CR2032) assembled in an argon-filled glove box. The working electrodes were prepared by coating the slurry consisting of 70 wt.% active materials, 15 wt.% Super P, and 15  wt.% poly (acrylic acid) (PAA, Mw = 3,000,000, Aldrich) dissolved in N-methyl pyrrolidinone (NMP) on copper foil. The cell was consisted of CuO/CNF as a positive electrode and Li foil as a counter electrode. The CuO/CNF was used as a working electrode by drying for 12 h at 130 °C in a vacuum oven to completely remove the water. The electrodes were separated by a separator (Celgard 2400). The electrolyte is composed of a solution of 1 M LiPF_6_ in a mixture of ethylene carbonate (EC)/dimethyl carbonate (DMC) (1:1, v/v) (Techno Semichem Co.). The Li foils were used a reference electrode and a counter one, respectively. Also, the charge-discharge performance of samples was measured by using a two-electrode system. The cyclic voltammetry (CV) test is carried out on an IM6e (Jahner Electrik IM6e, Germany) from 0 to 3 V. Electrochemical impedance spectroscopy (EIS) measurements were performed on IM6e (Jahner Electrik IM6e, Germany), and the frequency ranged from 10 m Hz to 100 kHz with an applied AC signal amplitude of 5 mV. The charge-discharge test was measured by using a battery cycler system (WBCS 3000, Won-A Tech. Co., Korea).

## Author Contributions

S.H.P designed the experiments, prepared the samples, performed the experiments, wrote the manuscript, and analyzed the data; W.J.L helped in writing the manuscript. All authors reviewed the manuscript.

## Additional Information

**How to cite this article**: Park, S.-H. and Lee, W.-J. Hierarchically mesoporous CuO/carbon nanofiber coaxial shell-core nanowires for lithium ion batteries. *Sci. Rep.* **5**, 09754; doi: 10.1038/srep09754 (2015).

## Figures and Tables

**Table 1:** Pore characteristics of CNF, CuO and CuO/CNF

	**Micropore surface area.(m**^**2**^** g**^**−1**^)	**Mesopore surface area.(m**^**2**^** g**^**−1**^)	**Total surface area (m**^**2**^** g**^**−1**^)	**Total pore volume (cm**^**3**^** g**^**−1**^)	**A.P.D^a^(nm)**	**Mesopore avg.dia^b^.(nm)**
CNF	367.1	11.6	378.7	0.17	1.82	3.92
CuO	0	5	5	0.02	43.82	15
CuO/CNF	186.3	58.4	244.7	0.46	7.57	29

^a^Average pore diameter.

^b^Calculated with 4 V/A of BJH method.

## References

[b1] Bruce P. G., Scrosati B. & Tarascon J. M. Nanomaterials for rechargeable lithium batteries. Angew. Chem. Int. Ed. 47, 2930–2946 (2008). 10.1002/anie.200702505 18338357

[b2] Poizot P., Laruelle S., Grugeon S., Dupont L. & Tarascon J. M. Nano-sized transition-metaloxides as negative-electrode materials for lithium-ion batteries. Nature 407, 496–499 (2000). 11028997 10.1038/35035045

[b3] Kim D. W., Ko Y. D., Park J. G. & Kim B. K. Formation of lithium-driven Active/Inactive nanocomposite electrodes based on Ca_3_Co_4_O_9_ nanoplates. Angew. Chem. Int. Ed. 46, 6654–6657 (2007). 10.1002/anie.200701628 17663491

[b4] Wang Z. Y., Luan D. Y., Madhavi S., Hu Y. & Lou X. W. Assembling carbon-coated alpha-Fe_2_O_3_ hollow nanohorns on the CNT backbone for superior lithium storage capability. Energ Environ. Sci. 5, 5252–5256 (2012).

[b5] Yin Z. G. & Zheng Q. D. Controlled Synthesis and Energy Applications of One-Dimensional Conducting Polymer Nanostructures: An Overview. Adv. Energy Mater . 2, 179–218 (2012).

[b6] Cui L. F., Shen J. A., Cheng F. Y., Tao Z. L. & Chen J. SnO_2_ nanoparticles@polypyrrole nanowires composite as anode materials for rechargeable lithium-ion batteries. J. Power Sources 196, 2195–2201 (2011).

[b7] Ko S., Lee J. I., Yang H. S., Park S. & Jeong U. Mesoporous CuO Particles Threaded with CNTs for High-Performance Lithium-Ion Battery Anodes. Adv. Mater. 24, 4451–4456 (2012). 22786742 10.1002/adma.201201821

[b8] Lu L. Q. & Wang Y. Facile synthesis of graphene-supported shuttle- and urchin-like CuO for high and fast Li-ion storage. Electrochem. Commun. 14, 82–85 (2012).

[b9] Zheng S. F. *et al.* Introducing dual functional CNT networks into CuO nanomicrospheres toward superior electrode materials for lithium-ion batteries. Chem. Mater. 20, 3617–3622 (2008).

[b10] Wang B., Wu X. L., Shu C. Y., Guo Y. G. & Wang C. R. Synthesis of CuO/graphene nanocomposite as a high-performance anode material for lithium-ion batteries. J. Mater. Chem. 20, 10661–10664 (2010).

[b11] Chen J. J. Recent Progress in Advanced Materials for Lithium Ion Batteries. Materials 6, 156–183 (2013). 10.3390/ma6010156 PMC5452126 28809300

[b12] Zhang R., Liu J., Guo H. & Tong X. Synthesis of CuO nanowire arrays as high-performance electrode for lithium ion batteries. Mater. Lett. 139, 55–58 (2015).

[b13] Yu Y., Chen C.H. & Shi Y. A tin-based amorphous oxide composite with a porous, spherical, multideck-cage morphology as a highly reversible anode material for lithium-ion batteries. Adv. Mater. 19, 993–997 (2007).

[b14] Liu X., Li Z., Zhang Q., Li F. & Kong T. Preparation of CuO/C core-shell nanowires and its application in lithium ion batteries. Mater. Lett. 80, 37–38 (2012).

[b15] Wang C. *et al.* Controlled synthesis of micro/nanostructured CuO anodes for lithium-ion batteries. Nano Energy 9, 334–344 (2014).

[b16] Wang J., Liu Y., Wang S., Guo X. & Liu Y. Facile fabrication of pompon-like hierarchical CuO hollow microspheres for high-performance lithium-ion batteries. J. Mater. Chem. A. 2, 1224–1229 (2014).

[b17] Verrelli R., Scrosati B., Sun Y.K. & Hassoun J. Stable, High Voltage Li_0.85_Ni_0.46_Cu_0.1_Mn_1.49_O_4_ Spinel Cathode in a Lithium-Ion Battery Using a Conversion-Type CuO Anode. ACS Appl. Mater. Interfaces 6, 5206–5211 (2014). 24611783 10.1021/am500499a

[b18] Zhou X. *et al.* Nanoleaf-on-sheet CuO/graphene composites: Microwave-assisted assemble and excellent electrochemical performances for lithium ion batteries. Electrochim. Acta 125, 615–621 (2014).

[b19] Liu Y. *et al.* Facile fabrication of CuO nanosheets on Cu substrate as anode materials for electrochemical energy storage. J. Alloy. Compd . 586, 208–215 (2014).

[b20] Zhang Y. *et al.* CuO Necklace: Controlled Synthesis of a Metal Oxide and Carbon Nanotube Heterostructure for Enhanced Lithium Storage Performance. J. Phys. Chem. C. 117, 12346–12351 (2013).

[b21] Zhang W. *et al.* Facile microemulsion synthesis of porous CuO nanosphere film and its application in lithium ion batteries. Electrochim. Acta 113, 63–68 (2013).

[b22] Huang H. *et al.* Self-assembly of mesoporous CuO nanosheets-CNT 3D-network composites for lithium-ion batteries. Nanoscale 5, 1785–1788 (2013). 23361121 10.1039/c3nr34070h

[b23] Xu Y., Jian G., Zachariah M. R. & Wang C. Nano-structured carbon-coated CuO hollow spheres as stable and high rate anodes for lithium-ion batteries. J. Mater. Chem. A. 1, 15486–15490 (2013).

[b24] Corni I., Ryan M. P. & Boccaccini A. R. Electrophoretic deposition: From traditional ceramics to nanotechnology. J. Eur. Ceram. Soc. 28, 1353–1367 (2008).

[b25] Wu M. S., Huang C. Y. & Lin K. H. Facile Electrophoretic Deposition of Ni-Decorated Carbon Nanotube Film for Electrochemical Capacitors. Electrochem. Solid State Lett. 12, A129–A131 (2009).

[b26] Santhanagopalan S., Teng F. & Meng D. D. High-voltage electrophoretic deposition for vertically aligned forests of one-dimensional nanoparticles. Langmuir 27, 561–9 (2011). 21171646 10.1021/la103587b

[b27] Hu L. *et al.* Silicon-Carbon Nanotube Coaxial Sponge as Li-Ion Anodes with High Areal Capacity. Adv. Energy Mater . 1, 523–527 (2011).

[b28] Wang J. W. *et al.* Sandwich-lithiation and longitudinal crack in amorphous silicon coated on carbon nanofibers. ACS. Nano 6, 9158–67 (2012). 22984869 10.1021/nn3034343

[b29] Sun S. *et al.* Hierarchical CuO nanoflowers: water-required synthesis and their application in a nonenzymatic glucose biosensor. Phys. Chem. Chem. Phys. 15, 10904–10913 (2013). 23698563 10.1039/c3cp50922b

[b30] Debart A., Dupont L., Poizot P., Leriche J. B. & Tarascon J. M. A transmission electron microscopy study of the reactivity mechanism of tailor-made CuO particles toward lithium. J. Electrochem. Soc. 148, A1266–A1274 (2001).

[b31] Grugeon S. *et al.* Particle size effects on the electrochemical performance of copper oxides toward lithium. J. Electrochem. Soc. 148, A285–A292 (2001).

[b32] Sahay R. *et al.* High Aspect Ratio Electrospun CuO Nanofibers as Anode Material for Lithium-Ion Batteries with Superior Cycleability. J. Phys. Chem. C. 116, 18087–18092 (2012).

[b33] Wang X. *et al.* Revealing the conversion mechanism of CuO nanowires during lithiation-delithiation by in situ transmission electron microscopy. Chem. Commun. 48, 4812–4814 (2012). 10.1039/c2cc30643c 22388332

[b34] Ming J. *et al.* The binder effect on an oxide-based anode in lithium and sodium-ion battery applications: the fastest way to ultrahigh performance. *Chem. Commun*. 50, 13307–13310 (2014). 10.1039/c4cc02657h 24934934

[b35] Park S. H., Jung H. R., Kim B. K. & Lee W. J. MWCNT/mesoporous carbon nanofibers composites prepared by electrospinning and silica template as counter electrodes for dye-sensitized solar cells. J. Photochem. Photobiol. A. 246, 45–49 (2012).

[b36] Park S. H., Jung H. R. & Lee W. J. Hollow activated carbon nanofibers prepared by electrospinning as counter electrodes for dye-sensitized solar cells. Electrochim. Acta. 102, 423–428 (2013).

[b37] Park S. H., Kim B. K. & Lee W. J. Electrospun activated carbon nanofibers with hollow core/highly mesoporous shell structure as counter electrodes for dye-sensitized solar cells. J. Power Sources 239, 122–127 (2013).

[b38] Battumur T. *et al.* Addition of multiwalled carbon nanotube and graphene nanosheet in cobalt oxide film for enhancement of capacitance in electrochemical capacitors. Curr. Appl. Phys. 13, 196–204 (2013).

